# Daily consumption of a mangosteen-based drink improves in vivo antioxidant and anti-inflammatory biomarkers in healthy adults: a randomized, double-blind, placebo-controlled clinical trial

**DOI:** 10.1002/fsn3.225

**Published:** 2015-04-13

**Authors:** Zhuohong Xie, Marsha Sintara, Tony Chang, Boxin Ou

**Affiliations:** International Chemistry Testing258 Main Street, Suite 202, Milford, Massachusetts

**Keywords:** Anti-inflammation, antioxidant, C-reactive protein, mangosteen, ORAC, *α*-mangostin

## Abstract

Mangosteen (*Garcinia mangostana*) is a tropical fruit cultivated mainly in Southeast Asia. Recent studies have shown mangosteen has many health benefits. In this study, we aimed to determine the effects of a mangosteen-based beverage on antioxidant and anti-inflammatory and immunity biomarkers in plasma of healthy adults. A randomized, double-blind, placebo-controlled clinical trial was conducted using 60 participants, 30 men, and 30 women, ages 18–60. Participants were randomly divided into two groups, placebo and mangosteen groups, with the same number of male and female participants in each group. The trial duration was 30 days. ORAC as an antioxidant biomarker was measured in both groups. It was found that after the 30-day trial, the group given the mangosteen-based drink formula showed 15% more antioxidant capacity in the bloodstream than did the placebo group. As for the inflammatory biomarkers, in the mangosteen group, between the preintervention and postintervention, the C-reactive protein level significantly decreased by 46%, while no significant decreases for the same biomarker was observed in the placebo group. Immunity biomarkers IgA, IgG, IgM, C3 and C4 were not affected in either group. In addition, the effects on hepatic function (Aspartate Aminotransferase and Alanine Aminotransferase) and kidney function (creatinine) were investigated. Our results indicated that after the 30-day consumption of the beverage, there were no side effects on human hepatic and kidney functions. The outcome of this study showed that the mangosteen-based formula significantly increases antioxidant capacity and possesses anti-inflammatory benefits with no side effects on immune, hepatic, and renal functions for long-term consumption.

## Introduction

Mangosteen is a tropical plant cultivated in areas such as Indonesia, Malaysia, Sri Lanka, Philippines, and Thailand. It has been consumed as fruit, juice and used as traditional medicine. Mangosteen has been used to treat skin infections and diarrhea. Recent scientific studies suggest that mangosteen possesses strong antioxidant, anti-cancer, anti-inflammatory, anti-allergic, anti-microbial, and anti-malarial properties (Gutierrez-Orozco and Failla 2013). Xanthone and vitamins in mangosteen are considered the major active components. Mangosteen extracts and xanthones from mangosteen were reported to scavenge 2,2-diphenyl-1-picrylhydrazyl (DPPH), 2,20-azino-bis-(3-ethylbenzthiazoline-6-sulfonic acid (ABTS), and peroxynitrite radicals (Yoshikawa et al. 1994; Jung et al. 2006; Haruenkit et al. 2007). Mangosteen was able to reduce LDL oxidation in vitro (Williams et al. 1995). Extracts of mangosteen protected against neural damage exposed to hydrogen peroxide on a neuroblastoma cell line (Weecharangsan et al. 2006). In a rat study, *α*-mangostin was able to attenuate the lipid peroxidation and damage of the antioxidant-defense system during injury-induced myocardial infarction (Sampath and Vijayaragavan 2008). In a clinical trial, we demonstrated a short-term increased antioxidant capacity and availability of mangosteen and vitamin B2 and B5 after a single dose oral administration of mangosteen drink (Kondo et al. 2009).

Chronic inflammation has been associated with cardiovascular diseases, cancer, diabetes, chronic lower respiratory disease, stroke, Alzheimer's disease, and nephritis. The annual death owing to these diseases was over 20 million worldwide in 2012 (WHO 2014). Chronic inflammation can be triggered by cellular stress and dysfunction, such as oxidative stress, elevated blood glucose levels, and excessive calorie consumption. Extracts and isolated compounds from mangosteen have been reported to attenuate proinflammatory response (Chairungsrilerd et al. 1996; Nakatani et al. 2002, 2004; Pongphasuk et al. 2003; Chen et al. 2008; Udani et al. 2009). *γ*-Mangostin has been reported to suppress inflammation in vitro by inhibiting the spontaneous PGE2 release and inhibit the expression of COX-2 (Nakatani et al. 2002, 2004). Chen et al. investigated the anti-inflammatory activity of *α*-manogstin and *γ*-mangostin and discovered that the activity was achieved through the inhibition of nitric oxide synthase (Chen et al. 2008). These two compounds were also found to block histamine H1 receptor in isolated rabbit thoracic aorta and guinea-pig trachea to block the effect of histamine, a regulator in the inflammatory response (Chairungsrilerd et al. 1996). Pongphasuk et al. discovered significant decrease of paw swelling in mice and albino rats in an inflammatory model (Pongphasuk et al. 2003). Udani et al. evaluated the mangosteen juice blend on biomarkers of inflammation in obese humans and found that C-reactive protein (CRP) was reduced (Udani et al. 2009). Gutierrez-Orozco and Failla summarized the current progress on anti-inflammatory research of mangosteen extracts, compounds, and products (Gutierrez-Orozco and Failla 2013). However, clinical trials focusing on anti-inflammatory effect of mangosteen are limited.

The evidence of health-promoting effectiveness of mangosteen is promising, which warrants our investigation on its long-term benefits in a clinical trial. In this study, we investigated the health effects of a functional beverage-containing mangosteen extract and other phytonutrients by measuring its impact on antioxidant status, inflammatory biomarker, and the immune responses in 60 health adults. The aim of this work was to determine the health benefits, as well as safety for consumption of mangosteen-based beverage.

## Materials and Methods

### Materials and reagents

*Trolox (6-hydroxy-2,5,7,8-tetramethyl-chroman-2-carboxylic acid), and Fluorescein sodium* were obtained from Sigma-Aldrich (St. Louis, MO)*,* 2,2'-Azobis (2-amidino-propane) dihydrochloride was purchased from Wako Chemicals USA (Richmond, VA). Verve® and placebo (fructose liquid) were provided by Vemma Nutrition Co. (Scottsdale, AZ). Verve® is a multivitamin/antioxidant liquid nutrition beverage containing mainly mangosteen fruit, a full spectrum of vitamins, green tea, aloe vera, and a caffeinated energy blend. Proprietary mangosteen juice and mangosteen extract, aloe vera gel, and green tea (decaffeinated) consisted of 10% wet weight of the beverage.

### Subjects and study protocol

A randomized, double-blind, placebo-controlled clinical trial was conducted with 60 generally healthy subjects (30 females and 30 males). Inclusion Criteria include: between 18 and 60 years of age; body mass index (BMI) between 20 and 30; willing to participate in the study; no supplementation of vitamins, minerals or antioxidants for at least 6 months; no history of long-term medication; and consumption of <2 cups of caffeinated beverage per day and abstaining from caffeine 24 h before each test day. The exclusion criteria include: existing heart, liver, lung, kidney or blood disease; allergies to mangosteen or other fruit juices; pregnancy or breastfeeding; previously or currently under anticoagulant therapy such as Coumadin; any acute or chronic medical or psychiatric condition; history of long-term medication; consumption of energy drinks (<2 weeks ago); and any acute or chronic medical or psychiatric conditions. All subjects were screened by evaluation of a medical history and assessment of diet history and supplement history using a self-developed semiquantitative questionnaire. Written informed consent was obtained from each volunteer participating in this study. All procedures of the protocol were approved by the Institutional Review Board of Hummingbird IRB (Cambridge, MA). Participants received a daily single dose (245 mL) of placebo (fructose liquid) or Verve energy drink (mangosteen-based formula). The use of fructose was to mimic the taste of Verve with minimal interference with absorption and availability of vitamins and phytonutrients. Participants fasted for 8 h before blood was drawn in the morning of day 1 and day 30. Physical measurements including body weight, BMI, blood pressure, and heart rate were conducted at each visit. Plasma was obtained by centrifugation and stored at −80°C until analysis.

### Antixoidant capacity of plasma (ORAC)

Plasma antioxidant capacity was determined by the ORAC assay as previously described using a Synergy H4 microplate florescence reader (Bio-Tek Instruments, Inc., Winooski, VT) with Gen 5V2 software (excitation wavelength 485 ± 20 nm, emission wavelength 530 ± 25 nm) (Ou et al. 2001; Prior et al. 2003). Plasma samples were thawed, vortexed, and centrifuged at16,000 g at 4°C for 3 min, 240 *μ*L of supernatant was deproteinized using 720 *μ*L of methanol. The mixture was vortexed for 30 sec, and then centrifuged at 16,000 g for 5 min at 4°C. Peroxyl radicals were generated by the spontaneous decomposition of AAPH at 37°C. Fluorescein was used as a fluorescent probe, with loss of fluorescence indicating fluorescein damage from its reaction with peroxyl radicals. The protective effects of the plasma tested were determined by comparing fluorescence time/intensity area under the curve (AUC) of the sample with a control.

### Inflammatory and immune biomarker analyses

Inflammatory marker C-reactive protein (CRP) was tested using a commercial immunoassay kit from Abcam (Cambridge, MA). Immunity biomarkers including Immunoglobulin (Ig) A, G, and M, complement C3, Complement C4, Interleukins (IL) 1-*α*, 1-*β*, and 2 in plasma were analyzed using commercial immunoassay kits from Abcam (Cambridge, MA) and eBioscience (San Diego, CA), respectively.

### Creatinine level and alanine transaminase (ALT) and aspartate aminotransferase (AST) activity

Urine creatinine levels were measured using a commercial kit from Abcam (Cambridge, MA). The ALT and AST activities were evaluated using commercial kits from Cayman (Ann Arbor, MI) and Abcam (Cambridge, MA) respectively.

### Statistical analysis

Data are reported as mean ± standard error (SEM). Student's t-test was employed to identify differences in means. Statistics were analyzed using SPSS for Windows (version rel. 10.0.5, 1999, SPSS Inc., Chicago, IL). Statistical significance was declared at *P* < 0.05.

## Results and Discussion

### Age, weight, body mass index, heart rate, systolic blood pressure (SBP) and diastolic blood pressure (DBP) of participants

The mean of ages, weight, body mass index (BMI), heart rate, systolic blood pressure, and diastolic blood pressure of participants are shown in Table1. As determined by Student's *t* test, anthropometric data were not significantly different between the mangosteen and the placebo groups and between before and after intervention. The intervention study design included 60 participants selected and randomly assigned into the mangosteen and placebo groups. The rationale of using 60 participants is to overcome the individual variability during ingestion of mangosteen product, as large discrepancy was seen in absorption rate of xanthone in mangosteen juice (Chitchumroonchokchai et al. 2012). All participants but three in the mangosteen group and one in the placebo group completed the study. The experimental data are analyzed based on those of the completed participants.

**Table 1:** Age, weight, BMI, heart rate, SBP and DBP of participants assigned to mangosteen and placebo groups before and after 30-day intervention

	Mangosteen		Placebo		*P*
*N*	27		29		
Age (years)	32.4 ± 1.7		31.5 ± 2.1		NS
	Before	After	Before	After	
Weight (kg)	79.0 ± 3.0	79.0 ± 2.8	73.7 ± 2.3	72.9 ± 2.2	NS
BMI (kg/m^2^)	26.5 ± 0.8	26.5 ± 0.7	25.5 ± 0.7	25.3 ± 0.6	NS
Heart rate (beats/min)	73.6 ± 2.2	72.0 ± 2.2	76.7 ± 2.4	70.0 ± 2.3	NS
SBP (mm Hg)	123.2 ± 2.2	117.7 ± 3.2	120.9 ± 1.9	121.0 ± 2.1	NS
DBP (mm Hg)	79.2 ± 1.8	74.6 ± 2.0	74.5 ± 1.4	74.9 ± 1.7	NS

Data are expressed as mean ± SEM. BMI, SBP and DBP stand for body mass index, systolic blood pressure and diastolic blood pressure respectively. The *P* value was calculated by unpaired two-tailed *t* test and NS represents no significant difference detected.

### Changes in antioxidant status

**Figure 1:**
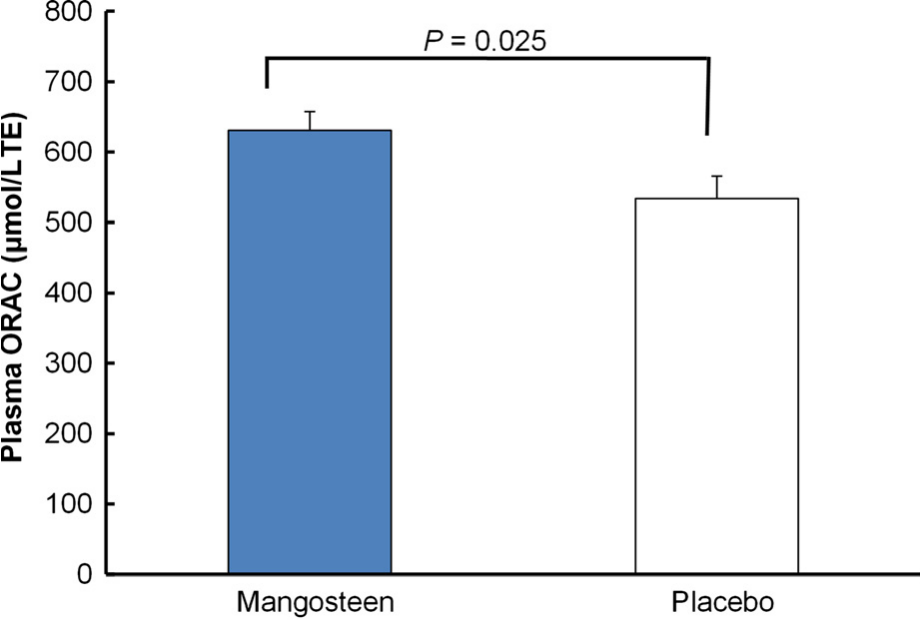
Plasma ORAC levels of mangosteen and placebo groups after intervention. Data are expressed as mean ± SEM. TE stands for Trolox equivalents. *P* value is calculated from comparing the means of groups using student's *t* test.

Xanthones are considered the major phytochemical compounds that have contributed to mangosteen's antioxidant capacity. Our recent studies have shown that xanthone-rich beverage effectively increased the peroxyl radical scavenging capacity (ORAC) of human plasma after a few hours of consumption (Kondo et al. 2009; Xie et al. 2015). As shown in Figure1, the antioxidant status of human plasma after 30 days was analyzed by hydrophilic ORAC, which accounts for activities of hydrophilic compounds. The antioxidant status in the mangosteen group was significantly higher compared to the placebo group. After the 30-day of intervention, the average ORAC value for mangosteen group was 630 *μ*mol/L, whereas the value for placebo group was 534 *μ*mol/L. The long-term increase in antioxidant level may be partly explained by the absorption and availability of bioactives from the beverage. These bioactives include xanthones, the major class of antioxidant in the beverage, along with phytochemicals from green tea and aloe vera and vitamin C and E. Xanthones have been reported to reach the peak level in plasma from one hour to 6 hours after ingestion (Kondo et al. 2009; Chitchumroonchokchai et al. 2012). In addition, xanthones and their metabolites were found to remain in plasma for as long as 24 h (Chitchumroonchokchai et al. 2012). The variability may be due to dosage, individual, and food matrix interaction. These bioactives may directly scavenge the free radicals in blood and attenuate the oxidative stress. In addition to the direct interaction with free radicals, more and more scientific evidence has suggested that phytochemicals, usually low at concentration in vivo, may operate by several mechanisms, such as elevation of the levels of endogenous antioxidant enzymes, including superoxide dismutase, catalase and glutathione peroxidase, interaction with in vivo metabolites, resulting in synergistic effect, or activation of the nrf2 pathway (Stevenson and Hurst 2007; Finley et al. 2011; Yang et al. 2014), which all account for explanation for the observed antioxidant health benefits.

### Changes in inflammatory biomarker C-reactive protein)

**Figure 2:**
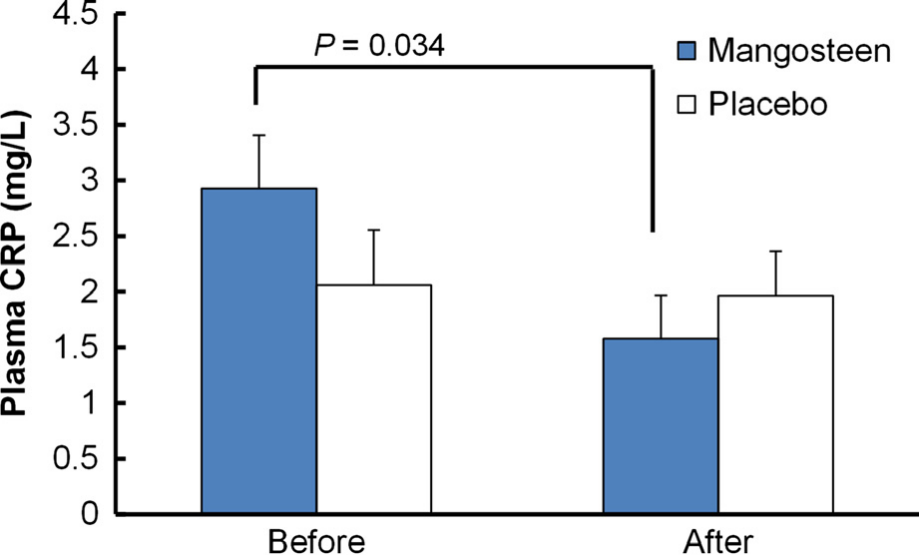
Plasma C-reactive protein concentrations of mangosteen and placebo groups before and after intervention. Data are expressed as mean ± SEM. *P* value is calculated from comparing the means of groups using student's *t* test.

Inflammation is linked with infections, diabetes, cancers, and cardiovascular diseases (Ridker et al. 2000; Freeman et al. 2002; Simon et al. 2004; Goyal et al. 2014). While inflammation is an intricate network of reactions, the level of C-reactive protein (CRP) is considered one of the measures to provide an overall estimate of inflammatory status. Even within the normal range, CRP in blood has served as an indicator for risks of chronic diseases (Ridker et al. 2000). The cohort study has suggested a lower level of CRP is associated with lower risk of colon cancer (Goyal et al. 2014). To examine the anti-inflammatory effect of the mangosteen-rich beverage, the CRP levels of 60 subjects were analyzed and displayed in Figure2. CRP levels between the treatment and placebo group were not statistically significant on day 1. CRP level in the mangosteen group was decreased significantly from 2.9 mg/L on day 1 to 1.6 mg/L on day 30 (*P* < 0.05, paired *t*-test) after consumption of the mangosteen product. The change of CRP level (<0.1 mg/L) in the placebo group was minimal and not statistically significant. The decrease in CPR level found in this study is consistent with a previous in vivo study in which Udani et al. found that the mangosteen product decreased CRP levels for 1–7% after an 8-week clinical study (Udani et al. 2009). The stronger effect seen in the current study might result from the dosage and synergistic effect of mangosteen, green tea, aloe vera, and vitamins. The significant decrease in CRP suggests that daily consumption of the mangosteen product might be able to lower the inflammatory status of healthy adults.

### Immunity biomarkers

**Figure 3:**
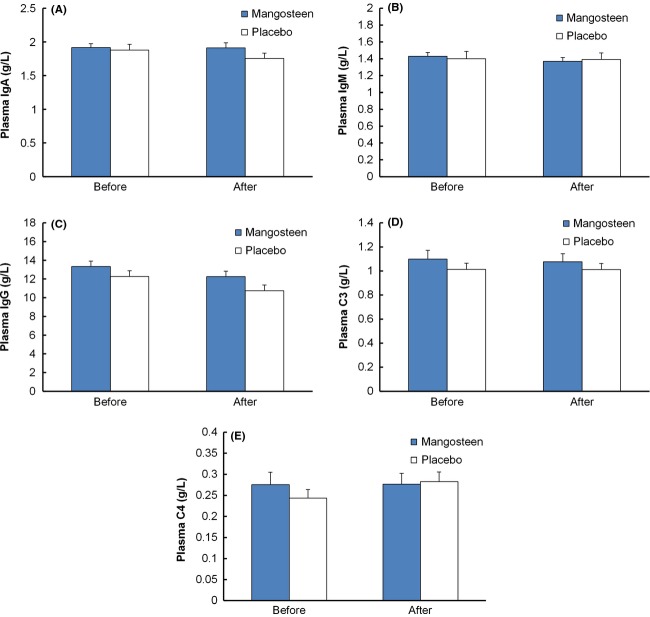
Plasma immunity biomarker concentrations of mangosteen and placebo groups before and after intervention: (A) Immunoglobulin A (IgA); (B) IgG; (C) IgM; (D) Complement 3 (C3); (E) C4. Data are expressed as mean ± SEM.

The immunity markers were also evaluated in this study. Immunoglobulins (Igs) are antibodies to identify and neutralize antigens such as bacteria and viruses in the immune system. As shown in Figure3, the Immunoglobulin A (IgA), IgM, and IgG in blood were determined. IgA in the mangosteen group had an average level of 1.9 g/L, whereas that in the placebo group was 1.8 g/L after intervention period. The difference is not significant (*P* = 0.158). Similarly, no statistical difference was observed in IgG and IgM after 30 days' beverage consumptions. The range for IgG of mangosteen group was 12.3 ± 0.6 g/L, while that of placebo group was 10.7 ± 0.6 g/L. The average level for IgM of both groups after intervention was 1.4 g/L. All antibodies tested were within the normal range, suggesting that the participants maintained a healthy state. Increased levels of Complement 3 (C3) and C4 may be one of the signs for inflammation. C3 and C4 levels in pre and postintervention were within the normal range. No significant difference was seen in immunoglobulin and complement proteins between different groups and between before and after the 30-day period, which indicates there is no adverse effect toward the immunity system after a 30-day consumption of mangosteen-rich beverage. Interleukin-1 (IL-1) and IL-2 are two classes of cytokines activated by pathogenic infections. IL-1*α*, -1*β,* and 2 were all in the normal range (0–5 ng/L) with no elevation found (data not shown). A wide range of botanicals were reported to have immunomodulatory effects (Tan and Vanitha 2004). Mangosteen-rich beverage overall did not change these markers. Immunomodulatory effects were not found in this study. On the other hand, these data implied no infections or severe inflammation was seen in subjects and mangosteen product did not exhibit significant effects on the Igs, complement proteins, and interleukins.

### Creatinine level and alanine transaminase (ALT) and aspartate aminotransferase (AST) activities

**Figure 4:**
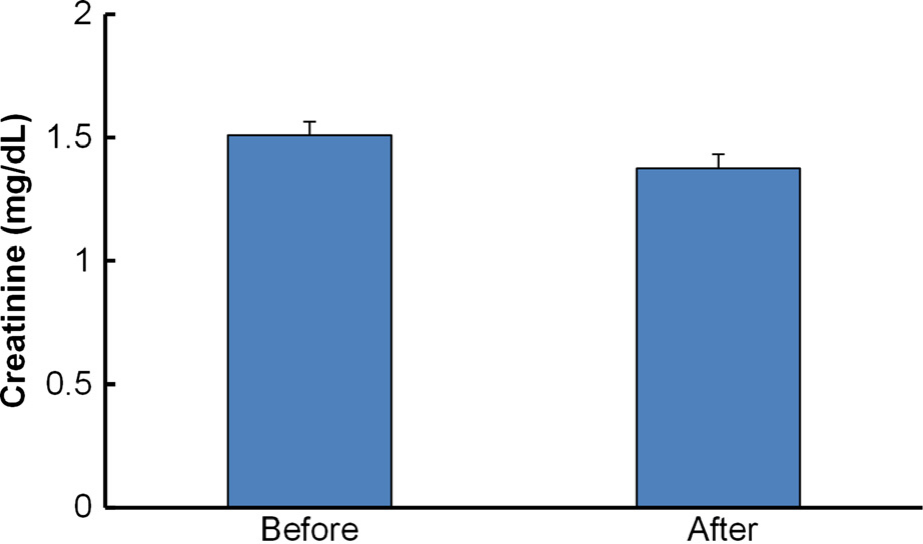
Creatinine concentration of mangosteen group before and after intervention. Data are expressed as mean ± SEM.

Creatinine is a waste compound generated from muscle metabolism of creatine phosphate. It is excreted unchanged by the kidney in human beings. The level of creatinine may be an indicator for overall renal health. Creatinine levels of mangosteen group after 30-day intervention period were analyzed by the enzymatic method and the result was shown in Figure4. No significant difference was observed between data from pre and postintervention. It suggests there is no adverse effect on kidneys after consumption of the mangosteen product.

**Figure 5:**
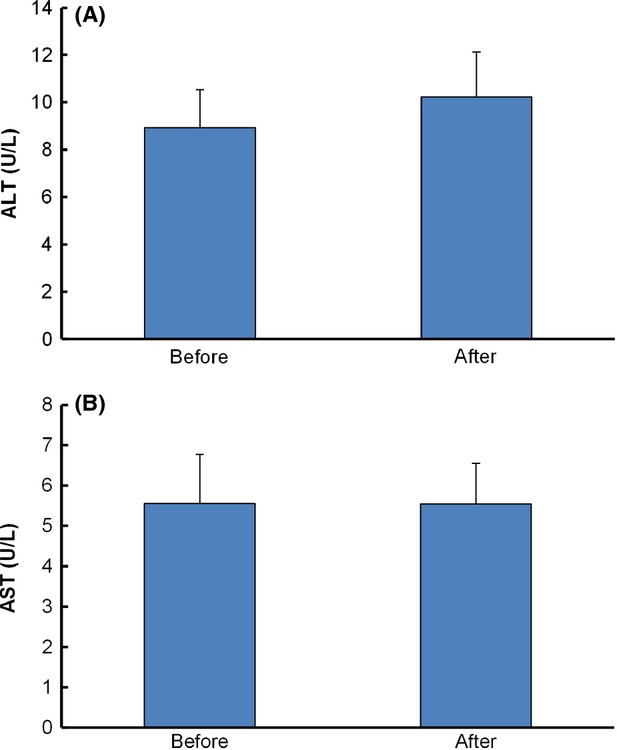
Alanine transaminase (ALT) and aspartate aminotransferase (AST) of mangosteen group before and after intervention. Data are expressed as mean ± SEM.

Alanine transaminase and AST are two enzymes normally found in the liver that are responsible for conversion of amino acids into glucose. The ALT and AST levels in blood are generally low except during disease status or tissue injury. The ALT and AST tests are usually used for evaluation of liver or other diseases (Vozarova et al. 2002; Kim et al. 2004). In the current study, ALT and AST levels of mangosteen group before and after 30 days of intervention are not significantly different (Fig.5). The data suggest that no adverse effect was seen in hepatic function after intervention with the mangosteen product.

In summary, for the first time, we demonstrated that 30-day ingestion of a mangosteen-rich energy drink increased the antioxidant capacity in human blood. The significant decrease in CRP level suggests a reduced risk of inflammation and related chronic diseases. Immunity markers including IgA, IgG, IgM, C3, C4, IL-1*α*, IL-1*β*, and IL-2 were not altered significantly by ingestion of the drink. The creatinine, ALT, and AST tests suggest no adverse effect from long-term consumption of the mangosteen product. Our results suggest that the mangosteen-rich energy drink is an excellent source to maintain balanced antioxidant status and can be part of the diet possibly against inflammation and chronic diseases. Further studies should explore the mechanisms on the in vivo antioxidant interactions with metabolites and the mediation of inflammation pathways.

## Conflict of Interest

The authors declare no conflict of interest.
